# Systems Biology Approach for Identification of Essential Growth Factors in Retinal Regeneration

**DOI:** 10.18502/jovr.v16i1.8242

**Published:** 2021-01-20

**Authors:** Zahra-Soheila Soheili, Hamid Latifi-Navid

**Affiliations:** ^1^Department of Molecular Medicine, National Institute of Genetic Engineering and Biotechnology, Tehran, Iran

Tissue engineering may be considered as a potential treatment modality for various types of retinal diseases. Retinal regeneration depends on an optimal combination of scaffolds, cells, and growth factors. Growth factors play a fundamental role in a variety of cellular processes such as proliferation, migration, differentiation, and multicellular morphogenesis.^[1]^ Growth factors expedite tissue growth/regeneration by providing the right signals to the cells.^[2]^ Systems biology-related approaches help us understand the mechanisms underlying retinal tissue engineering and investigate the effect of growth factors through protein–protein interaction network analyses. Network centrality analysis using different criteria has the potential to reveal growth factors important for retinal regeneration.

In the current issue of *Journal of Ophthalmic and Vision Research*, Beheshtizadeh *et al* report an *in-silico *study which was aimed to determine the most important growth factors in retinal tissue engineering.^[3]^ Gene ontology (GO) and degree centrality analysis were used to identify the most effective proteins for retinal regeneration. Despite the remarkable results presented in the study, it should be stressed that the growth factors were determined only by degree centrality analysis. Numerous studies have established that low connectivity growth factors may also be considered critical in biological processes and for network integrity.^[4,5]^

There are several types of centrality which include degree, closeness, between-ness, centroid value, bridging, eccentricity, and eigenvector centrality. Degree centrality is used to evaluate the regulatory importance of immediate neighboring nodes. Nodes with high degree centrality interact with different proteins and therefore usually play a key regulatory role in the network. The short average distance of a distinct node to the entire network of proteins is represented by the closeness index. Proteins with high closeness index (compared to the network) impose a fundamental regulatory effect on other proteins and will be significantly affected by changes in the network. Between-ness index represents the number of times that a specific node (via the shortest path) is used to hold communicating proteins together. The coherence and functionality of the network are likely maintained by the betweenness centrality index. To determine the functional ability of a distinct node to orchestrate discrete clusters of proteins, centroid value is used in the network. Nodes with high centroid values coordinate the activity of other clusters to regulate a distinct cell function. Bridging centrality index is employed to distinguish nodes that link clusters or densely connected regions. Eccentricity index is used to distinguish nodes which are easily reachable by all other proteins. Therefore, a protein with high eccentricity index affects, or gets more easily affected by, other proteins. To determine nodes with a central super-regulatory role or those that serve as key targets of a regulatory pathway, the eigenvector centrality index is used.^[6]^

In summary, to comprehensively understand the importance of each node in any given network, different kinds of centrality analyses should be performed. Moreover, integration of centrality analyses results helps one correct selection of the most important nodes in the network. It is highly recommended to use web servers such as DAVID (Database for Annotation, Visualization, and Integrated Discovery; https://david.ncifcrf.gov/) or Enrichr (https://maayanlab.cloud/Enrichr/) which have been specifically developed for gene ontology (GO) and enrichment analyses.^[7,8]^ Moreover, there exists a need for developing a software which integrates servers that test for GO category enrichment via recruiting the output and provide the resources for summarizing and visualizing data.
